# Rehabilitating the cyanobacteria – niche partitioning, resource use efficiency and phytoplankton community structure during diazotrophic cyanobacterial blooms

**DOI:** 10.1111/1365-2745.12437

**Published:** 2015-07-02

**Authors:** Kalle Olli, Riina Klais, Timo Tamminen

**Affiliations:** ^1^ Institute of Ecology and Earth SciencesUniversity of TartuLai 4051005TartuEstonia; ^2^ Marine Research CentreFinnish Environment InstituteP.O. Box 14000251HelsinkiFinland

**Keywords:** *Aphanizomenon*, aquatic plant ecology, Baltic Sea, community assembly, functional diversity, nitrogen fixation, *Nodularia*, nutrient limitation, phylogenetic diversity

## Abstract

1. Blooms of nitrogen‐fixing cyanobacteria are recurrent phenomena in marine and freshwater habitats, and their supplying role in aquatic biogeochemical cycles is generally considered vital. The objective of this study was to analyse whether an increasing proportion of nitrogen‐fixing cyanobacteria affects (i) the composition of the non‐diazotrophic component of ambient phytoplankton communities and (ii) resource use efficiency (RUE; ratio of Chl *a* to total nutrients) – an important ecosystem function. We hypothesize that diazotrophs increase community P use and decrease N use efficiencies, as new N is brought into the system, relaxing N, and concomitantly aggravating P limitation. We test this by analysing an extensive data set from the Baltic Sea (> 3700 quantitative phytoplankton samples), known to harbour conspicuous and recurrent blooms of *Nodularia spumigena* and *Aphanizomenon* sp.
2. System‐level phosphorus use efficiency (RUE_P_) was positively related to high proportion of diazotrophic cyanobacteria, suggesting aggravation of phosphorus limitation. However, concomitant decrease of nitrogen use efficiency (RUE_N_) was not observed. *Nodularia spumigena*, a dominant diazotroph and a notorious toxin producer, had a significantly stronger relationship with RUE_P_, compared to the competing non‐toxic *Aphanizomenon* sp., confirming niche differentiation in P acquisition strategies between the major bloom‐forming cyanobacterial species in the Baltic Sea. *Nodularia* occurrences were associated with stronger temperature stratification in more offshore environments, indicating higher reliance on *in situ* P regeneration.
3. By using constrained and unconstrained ordination, permutational multivariate analysis of variance and local similarity analysis, we show that diazotrophic cyanobacteria explained no more than a few percentage of the ambient phytoplankton community variation. The analyses furthermore yielded rather evenly distributed negative and positive effects on individual co‐occurring phytoplankton taxa, with no obvious phylogenetic or functional trait‐based patterns.
4. *Synthesis*. Our study reveals that despite the widely acknowledged noxious impacts of cyanobacterial blooms, the overall effect on phytoplankton community structure is minor. There are no predominantly positive or negative associations with ambient phytoplankton species. Species‐specific niche differences in cyanobacterial resource acquisition affect important ecosystem functions, such as biomass production per unit limiting resource.

## Introduction

Nitrogen‐fixing microbes are an essential source of biologically available N in ecosystems, affecting the productivity of N‐limited communities by compensating for nitrogen losses via denitrification and anammox (Moisander *et al*. 2010; Zehr & Kudela 2011). Marine environments are generally N‐limited, and therefore, microbial N_2_ fixers (diazotrophs) are considered to play a central role in their biogeochemical cycles and maintaining the stoichiometric homoeostasis (Howarth 1988; Vitousek & Howarth 1991).

In the global ocean, N_2_ fixation is carried out by several functional types of diazotrophs. Most important types are the non‐heterocystous filamentous cyanobacteria (e.g. *Trichodesmium* spp.), unicellular cyanobacteria (e.g. *Crocosphaera watsonii* and uncultured cyanobacteria related to *Cyanothece* sp.), heterocystous filamentous forms and symbiotic diazotrophs associated with diatoms (Monteiro, Follows & Dutkiewicz 2010; Sohm, Webb & Capone 2011; Zehr 2011). In general, diazotrophs grow slower than other phytoplankton and they require more iron (Sohm, Webb & Capone 2011). They will be out‐competed if dissolved inorganic phosphorus (P) or iron (Fe) limits them and their non‐diazotroph competitors (Dutkiewicz, Ward & Scott 2014). Therefore, the global biogeography of diazotrophs, as well as N_2_ fixation rates, can be understood as a function of temperature, and excess supply of both iron and phosphorus over nitrogen (Monteiro, Follows & Dutkiewicz 2010; Monteiro & Dutkiewicz 2011). Current oceanic nitrogen fixation estimates (100–150 Tg N per year), representing ca. 47% of the total nitrogen sources to the global marine nitrogen budget (Gruber & Sarmiento 1997; Monteiro, Follows & Dutkiewicz 2010), correspond to half of the global biological N_2_ fixation (Canfield, Glazer & Falkowski 2010).

In the landlocked Baltic Sea, the Fe and other nutrients supplied by terrestrial run‐off support extremely high rates of N_2_ fixation, 55–840 mmol N m^−2^ year^−1^, corresponding to as much as 55% of the annual N input (Larsson *et al*. 2001; Wasmund *et al*. 2005; Stolte *et al*. 2006). The regular nuisance blooms in the Baltic Sea are caused by the heterocystous filamentous‐type diazotrophs, mainly by *Nodularia spumigena* and *Aphanizomenon* sp. (Bianchi, Engelhaupt & Westman 2000; Finni *et al*. 2001; Kahru & Elmgren 2014). For brevity, both taxa are referred by their genus names below. Experimental evidence indicates important niche differences between *Nodularia* and *Aphanizomenon*. *Aphanizomenon* is of freshwater origin and prefers lower salinity (0–5) and temperature (16–22 °C) compared to *Nodularia* (optima at salinity 5–10, temperature 25–28 °C) (Lehtimäki *et al*. 1997; Hajdu, Höglander & Larsson 2007). *Nodularia* is considered a harmful cyanobacterium because it produces a hepatotoxic pentapeptide called nodularin (Sivonen *et al*. 2007). The Baltic Sea strain of *Aphanizomenon* is considered non‐toxic, despite the toxicity of several congeneric freshwater species (Lehtimäki *et al*. 1997).

Cyanobacterial blooms are increasing globally, and since they can benefit from climate change and anthropogenic activities in several ways (O'Neil *et al*. 2011; Paerl & Paul 2012), they are likely to continue their increase in the future. Bloom‐forming cyanobacteria are strongly favoured by elevated water temperature and irradiance and by a stable water column (Kononen *et al*. 1996; Paerl & Otten 2013). The projected climate warming and increasing frequency of extreme heat periods (Paul 2008; O'Neil *et al*. 2011) potentially exacerbate the environmental problems caused by cyanobacterial blooms. Recent global modelling studies predict that warmer and dustier climates favour diazotrophs due to an increase in the ratio of supply rate of iron to fixed nitrogen (Monteiro, Follows & Dutkiewicz 2010; Dutkiewicz, Ward & Scott 2014). Realistic scenarios of climate change‐driven alterations in ocean temperature, circulation, mixing and increases of dustborne iron supplies suggest 17–38% increase in the area of global ocean sustaining diazotrophs and total global nitrogen fixation rates (Dutkiewicz, Ward & Scott 2014).

In the Baltic Sea, profiles of cyanobacteria‐specific pigments in the sediments suggest their presence throughout millennia, but a dramatic increase since late 1960s (Bianchi, Engelhaupt & Westman 2000; Poutanen & Nikkilä 2001). A common perception is that cyanobacterial blooms in the Baltic Sea have increased in the recent decades (Finni *et al*. 2001; Suikkanen, Laamanen & Huttunen 2007), but whether the increase reflects anthropogenic influence is a question still debated among the scientists and general public (Bianchi, Engelhaupt & Westman 2000; Zillén & Conley 2010). A compilation of a 35‐year‐long satellite‐based time series (1979–2013) of cyanobacterial surface accumulations in the Baltic Sea does not confirm long‐term increase, but rather decadal‐scale oscillations (Kahru & Elmgren 2014).

The harmful aspects of cyanobacterial blooms include the loss of water clarity, suppressing other aquatic phototrophs and macrophytes, as well as their contribution to bottom‐near oxygen consumption, and negative effects on higher trophic levels including fish (Karjalainen *et al*. 2007; Paerl & Otten 2013). Direct and indirect toxic or allelopathic effects of filamentous cyanobacterial blooms on individual species of several trophic levels have been repeatedly studied experimentally (e.g. Sellner, Olson & Olli 1996; Suikkanen 2004; Engström‐Öst *et al*. 2011). Toxins are transferred both in planktonic and benthic food chains, for example filter feeders and benthic fish accumulate toxins in soft tissues, posing a potential risk for higher trophic levels and humans (Sipiä *et al*. 2001; Sopanen *et al*. 2009; Karlson & Mozūraitis 2011). These predominantly negative perspectives contrast the major ecosystem service provided by diazotrophic cyanobacteria – maintenance of homoeostasis and nutrient stoichiometry in the global ocean (Deutsch *et al*. 2007). However, next to nothing is known about how natural blooms of diazotrophic cyanobacteria affect the structure of the rest of the phytoplankton community and the community‐level resource use patterns. Experimental evidence suggests that N‐fixing cyanobacteria induce ecosystem‐level phosphorus limitation and favour specific types of co‐occurring phytoplankton (Kangro *et al*. 2007; Seppälä & Olli 2008).

Despite the high energetic cost of N_2_ fixation, dissolved bioavailable nitrogen release from cyanobacteria is in the range of 40–80% of the rate of N_2_ fixation (Ohlendieck *et al*. 2007; Wannicke, Koch & Voss 2009; Ploug *et al*. 2011). The released new nitrogen is readily redistributed throughout the food web, from the picoplanktonic fraction to mesozooplankton (Mulholland 2007; Wannicke *et al*. 2013). Resource supply ratios are instrumental to determine winners and losers (Tilman 1977; Dutkiewicz, Ward & Scott 2014); therefore, relaxation of N limitation could shift the structure and species composition of a phytoplankton community.

The aim of this study was to investigate whether, and to what extent, diazotrophic cyanobacteria blooms affect the ambient phytoplankton community structure and composition, and whether cyanobacterial blooms are reflected in an essential ecosystem function, the resource use efficiency of the community. We studied this by analysing an extensive phytoplankton community data set, which was merged from decadal monitoring time series by nine academic institutions during 1966–2008, covering the major basins of the Baltic Sea (Olli *et al*. 2013).

We hypothesized that intensifying diazotrophic cyanobacterial blooms have a systematic and directional effect on the non‐diazotrophic phytoplankton community. We further postulated that an increasing proportion of diazotrophic cyanobacteria provides a new nitrogen source to the nitrogen‐limited summer plankton community. We therefore hypothesized that the diazotrophic nitrogen import alleviates prevailing nitrogen limitation (Tamminen & Andersen 2007), concomitantly aggravating phosphorus limitation. The shift from N to P limitation would thus correlate with the variation in resource use efficiency (RUE), an important ecosystem process affecting the biomass yield of phytoplankton community per unit limiting nutrient (Ptacnik *et al*. 2008). We consequently hypothesized that phosphorus use efficiency (RUE_P_) increases and nitrogen use efficiency (RUE_N_) decreases along a gradient of increasing cyanobacterial dominance.

## Material and methods

The data analysis consists of four blocks. First, we use three complementary community analysis techniques to study how much of the variation in the non‐diazotrophic component of the phytoplankton assemblage is explained by the diazotrophic cyanobacteria in the communities. Secondly, we apply regression analysis to study whether variation in the diazotroph proportion is associated with resource use efficiency. Thirdly, we use local similarity analysis to detect species in the community, which recurrently show positive or negative co‐occurrence patterns with diazotrophs. Finally, we track potential functional trait and phylogenetic signals in the co‐occurrence patterns. We test whether positive or negative associations with diazotrophs converge at groups of taxa with close functional trait or phylogenetic similarity, that is whether the species associations with diazotrophs show phylogenetic or trait‐based autocorrelation.

### Data set

Quantitative phytoplankton and environmental time series (1966–2008) were harmonized from environmental monitoring data sets of seven Baltic Sea countries (Olli *et al*. 2013). All analyses were restricted to the surface mixed layer, and to the summer season, when cyanobacterial blooms are frequently observed: from July to September in the southern Baltic Sea and from August to September in the northern Baltic Sea and the Gulfs of Finland and Riga. To account for large‐scale spatial heterogeneity, all community analyses were done separately for the three major Baltic Sea basins with major cyanobacterial blooms: the Gulf of Finland, the Gulf of Riga and the Baltic Proper. Cyanobacterial blooms are uncommon in the northern basin – the Gulf of Bothnia.

Diazotrophs were defined as species belonging to the heterocyst possessing genera *Aphanizomenon*,* Nodularia*,* Dolichospermum* and *Anabaenopsis*. Bloom intensity was defined as the proportion of diazotrophic biomass from the total phytoplankton wet weight biomass in the sample. The analyses were repeated with log‐transformed diazotrophic biomass, as a measure of absolute bloom magnitude instead of relative dominance. The results generally agreed with the proportion approach and are presented only when the two complement each other. We further detailed the analysis by considering separately *Aphanizomenon* and *Nodularia*, the two main bloom‐forming cyanobacteria in the Baltic Sea.

The Baltic Sea phytoplankton summer community has gone through a pronounced multidecadal temporal shift in compositional structure, which strongly affects ordination topology (Olli *et al*. 2011). To reduce the effect of this long‐term change, we restricted the community analysis to two decades – 1994–2004.

### Community analysis

Observational community ecology data frequently contain much redundant information: species often share similar distributions. Here, we use two community ordination techniques to reduce the redundancy of the original data and to represent important and interpretable environmental gradients. In unconstrained ordination, external variables do not intervene in the calculation of the community ordination. Correlation of the ordination vectors on environmental variables is computed *a posteriori*. On the contrary, in constrained analysis, *a priori* defined external variables intervene in the calculation, constraining the ordination vectors to be maximally related to combination of the external variables, bearing some similarity with multiple regression (Legendre & Legendre 1998). Ordination analysis in reduced space inevitably omits some portion of the ecological information, and any statistical inference would be limited to points in the first few dimensions, rather than being applicable to the original observations. To analyse the community–diazotroph relation in full space, we use permutational multivariate analysis of variance. This is a nonparametric analogue of manova but does not assume multivariate normality, which is usually unrealistic with highly aggregated species abundance distributions.

We used non‐metric multidimensional scaling (NMDS, *metaMDS* in R package *vegan*), which is a distance‐based approach considered as the most robust unconstrained ordination method in community ecology (Legendre & Legendre 1998). The community data (samples in rows and taxa in columns) contained phytoplankton biovolumes, excluding the diazotrophs and rare species (present in three of fewer samples). The data matrix was square‐root‐transformed to stabilize variability, followed by Wisconsin double standardization (first, species divided by their maxima; second, samples divided by sample totals). From the standardized community matrix, a Bray–Curtis dissimilarity matrix was calculated and subjected NMDS. NMDS projects the observed dissimilarities onto a two‐dimensional ordination space, and it can handle nonlinear species responses.

Proportion of the nitrogen fixers was fitted onto the ordination plane as an external variable, to visualize the direction and strength of the most rapid change in the sample diazotrophs within the ordination space. The strength of the association was measured by the square of the correlation coefficient (*R* ^2^) between the ordination and the variable; the statistical significance of the association (*P*‐value) was assessed by randomizing diazotroph proportions between the samples (999 simulations). If a similar or higher *R* ^2^ was frequently obtained with random permutations, the association of the diazotrophs with the community ordination was considered insignificant.

We further used constrained correspondence analysis (*cca* in R package *vegan*), an eigenanalysis‐based ordination method that partitions the variation in community composition into components that can be explained by external variables (i.e. the diazotroph proportion), and an unconstrained component. The significance of the external variable on community composition was assessed by a permutation test. Diazotroph proportion was randomized among samples, and the model was refitted. When the explained variation in the permutations was lower than that observed originally, the effect of diazotrophs was considered statistically significant.

Cyanobacterial bloom intensity correlates with a variety of environmental factors, such as elevated water temperature, radiation, slack winds, seasonal timing and distance from the shore. It is thus not immediately obvious whether the gradients in community composition are due to the effect of high proportion of diazotrophic biomass, or due to the correlating environmental factors alone. We therefore performed a partial constrained ordination, where the effect of the background environmental variables was removed before constraining the ordination with the proportion of diazotrophs. If the constrained variance decreases after adding the conditional environmental variables, then part of the constrained variance initially attributed to diazotrophs could have been due to the background variables.

Permutational multivariate analysis of variance, a nonparametric analogue of manova, was used as a third approach to assess the effect of diazotrophs on the community variation (*adonis* in R package *vegan*). The method partitions sums of squares using semi‐metric and metric distance matrices and is a robust alternative to ordination methods for describing how variation in community composition is attributed to different external covariates. Although this is not an ordination method, for the sake of comparability, we used also here a temporally restricted data set (1994–2004).

### Community resource use efficiency as a function of cyanobacterial bloom intensity

We tested whether there is an association between the proportion of diazotrophs and the resource use efficiency of phytoplankton communities. We defined phytoplankton resource use efficiency as a natural logarithm of the weight ratio between biomass, estimated as chlorophyll a (Chl *a*), and total nitrogen (TN), or total phosphorus (TP) concentrations (Ptacnik *et al*. 2008).

$${\text{RUE}}_{P}=\log\left(\text{Chl}\;a/\text{TP}\right);{\text{RUE}}_{N}=\log\left(\text{Chl}\;a/\text{TN}\right)$$

By estimating RUE from chemical variables (Chl *a*, TN, TP), RUE is methodologically independent from the microscopy‐based plankton community analysis. Before regression analysis, we normalized both RUE_P_ and RUE_N_ to their respective z‐scores, having zero mean and unit standard deviation. Normalization brings both species of RUE to the same scale and enables comparison of linear regression slopes. The proportion of diazotrophs was arcsine square‐root‐transformed before analysis. The relationship between RUE and proportion of diazotrophs was analysed with general additive models (*gam* in R package *mgcv*), with geographic location as a nonparametric smooth term and asin square‐root‐transformed proportion of diazotrophs as a linear term. RUE has a notable geographic pattern in the Baltic Sea, related to a shift in nutrient limitation patterns along the natural salinity gradient (Tamminen & Andersen 2007). To account for this non‐random background, we added the geographic location (latitude and longitude) to the model as a nonparametric covariate.

### Local similarity analysis

We used local similarity analysis (LSA) to assign phytoplankton species into groupings that tend to co‐occur and those that tend not to co‐occur with high proportion of diazotrophs. LSA is a mathematical method designed to detect co‐occurrences between organisms, as well as between organisms and environmental variables (Ruan *et al*. 2006). The method is particularly well suited for the analysis of temporal sequences and differs from Pearson correlation by taking into account co‐occurrence patterns with time delay, and associations which occur within subintervals of the data set (see Ruan *et al*. 2006). For LSA, we used community data matrices, amended with columns with the diazotrophic biomass and proportions in the sample, and normalized by rank normal score transformation. The resulting local similarity scores between pairs of species have a theoretical range between −1 and 1, and they can be interpreted similarly to correlation coefficients. Positive or negative LS scores designate positive or negative co‐occurrence links between pairs of species, or between species and external variables. Here, we were interested in which species had positive or negative connections with the diazotrophs. LSA takes advantage of the time‐series nature of the data, and the values of the LS scores therefore depend on the order of samples in the time series. Our data set had large spatial and temporal variation and did not conform to the strict format of a regular time series. Thus, we did not use the time‐series merit of LSA; instead, we required the LS scores to be independent of the order of samples in the community matrix. To break down the time‐series structure, we randomized the order of samples 1000 times for each LSA and used the average LS scores as an association parameter. For the significance of the association parameter, we used the conservative requirement that all the individual randomized analyses had to be either positive or negative.

### Functional trait and phylogenetic signals in the association between species and the diazotrophs

The LS scores quantify the positive or negative associations between the species and the diazotrophs. We studied whether these associations are non‐random within the functional trait space or within the phylogenetic hierarchy. We hypothesized that there are phylogenetically closely related taxa, which tend to have more often positive (or negative) associations with high diazotroph proportion than expected by random chance. Equivalently, we test whether taxa, which have positive or negative association with diazotrophs, share common functional traits, that is are functionally more similar than expected by chance alone.

We assigned functional trait values to the taxa in the community matrices. These included qualitative binary traits – auxiliary pigment composition, trophic state (auto‐, hetero‐, mixotrophy), motility, silica uptake, and colony or chain formation. We also used cell (or colony) size as a quantitative trait. From the taxa × traits matrix, we calculated Gower distances for each taxon pair. The test statistic was the mean weighted pairwise distance (MPD, *mpd* in R package *picante*) between the observed taxa in each sub‐basin. Weights were the cross products of the LS scores between the respective taxa and diazotrophs. Weights can only be non‐negative; thus, a constant was added to rescale all LS scores to positive values. Thus, if taxa, which are positively associated with cyanobacteria and thus have high LS scores, tend to share common functional traits, MPD would be low (trait convergence). MPD was evaluated against a null model, where the LS scores were randomized between the species. The number of times that the observed MPD value was lower than the randomized MPD value indicated the strength of trait similarity between those species, which were positively associated with high diazotroph proportion. Concomitantly, observed MPD higher than the randomized MPD indicated functional trait similarity among those species negatively associated with diazotrophs.

For phylogenetic diversity analyses, we used the taxonomic topology in the World Register of Marine Species (WoRMS, Costello *et al*. 2013). The high resolution of WoRMS topology (including the main taxonomic ranks, as well as secondary ranks) was essential when characterizing the phylogenetic structure of organisms belonging to the polyphyletic assemblage known as phytoplankton. Only the topology information and not the branch lengths were considered, as such information is not widely available. The phylogenetic distance between two taxa was defined as the intergroup dissimilarity at which the two taxa are first combined into a single cluster. As with functional diversity, the test statistic was the MPD separating taxa in the regional species pool. As above, weights were the cross products of the rescaled LS scores, and the observed MPD was evaluated against a null model, where the LS scores were randomized between the species.

Randomized MPD has an approximately normal distribution. Subtracting mean randomized MPD from the observed MPD, and dividing by the standard deviation of randomized MPD, gives the standardized effect size (SES, ses.*mpd* in R package *picante*). Because SES is a standard normal deviate with zero mean and unit standard deviation, it is a suitable metric for comparisons across ecosystems and organism groups. Negative SES values indicate the prevalence of phylogenetic or functional convergence among species positively affected by diazotrophs. Positive SES suggests phylogenetic or functional convergence among species is negatively affected by diazotrophs. SES outside the range of −2 to 2, that is outside ±2 standard deviations, strongly suggests non‐randomness of the observed MPD.

## Results

**Figure 1:**
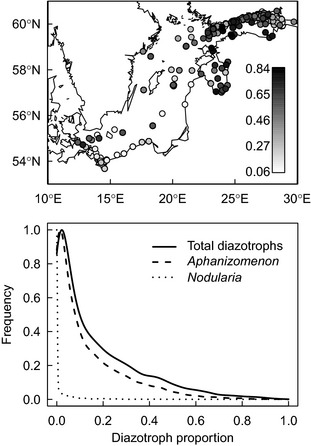
Spatial distribution of phytoplankton summer samples in the data set (upper panel). The grey scale of the symbols reflects the proportion of the diazotrophic biomass in the sample. The symbols are plotted in the order of increasing diazotrophic proportion and in overlapping symbols, high‐proportion samples overlay low‐proportion samples. Frequency distribution of the proportions of total diazotrophs, *Aphanizomenon* sp and *Nodularia spumigena* biomass in the samples (lower panel). Note the strongly skewed distributions.

After seasonal constraining, the data set contained 3761 quantitative summer phytoplankton samples from the surface mixed layer. The spatial sample distribution was aggregated and biased towards coastal zone (Fig. 1a). The mean proportion of diazotrophs biomass in the samples was 0.17 and had a high variability (range 0–0.96; Table 1). The frequency distribution was strongly positively skewed, which was particularly pronounced for *Nodularia* (Fig. 1b). The biomass proportions of *Aphanizomenon* and *Nodularia* had only a weak positive correlation, though, statistically significant due to the large sample size (Pearson correlation coefficient = 0.16, *P* < 10^−16^, *n* = 3761).

**Table 1:** The mean (±SD), median and maximum proportion of total diazotrophic biomass, and the proportions of the two main species, *Nodularia spumigena* and *Aphanizomenon* sp. in the 3761 Baltic Sea summer samples

	Total diazotrophs	*Nodularia*	*Aphanizomenon*
Mean ± SD	0.17 ± 0.19	0.03 ± 0.07	0.13 ± 0.15
Median	0.10	0	0.07
Max	0.96	0.75	0.95

### Diazotroph niche separation along environmental and spatio‐temporal gradients

We used weighted average and randomization to test spatio‐temporal and environmental niche separation between *Nodularia* and *Aphanizomenon*. The average water temperature, weighted by the biomass proportions of *Nodularia* (17.3 °C), was 1 °C higher compared to weighting with *Aphanizomenon* biomass proportion (16.3 °C). The difference was significantly higher (*P* < 0.01, *n* = 2618) than expected by chance and was verified by randomly shuffling *Nodularia* and *Aphanizomenon* proportions within the samples, followed by recalculating the difference of weighted averages 999 times. The weighted mean salinity difference was small (0.08), providing some support to the higher salinity preference of *Nodularia* (*P* = 0.08 with proportions, *P* = 0.01 with biomasses, *n* = 2559).

The field data revealed a 7 km difference in the weighted mean distance from the shoreline (*P* < 0.01, *n* = 3761), suggesting spatial decoupling between the more pelagic *Nodularia* compared to the more coastal *Aphanizomenon*. However, there was only a weak or no statistical support (*P* = 0.04 with proportions, *P* = 0.3 with biomasses, *n* = 3761) for a 2‐day temporal delay of *Aphanizomenon* compared to *Nodularia*. Thus, despite considerable overlap, niche differences were mostly significant. The field data provide a strong support for the experimental evidence of higher temperature preference of *Nodularia* compared to *Aphanizomenon* and a weaker support for the higher salinity preference (Lehtimäki *et al*. 1997; Hajdu, Höglander & Larsson 2007).

### Community P use efficiency scales with diazotroph proportion

The proportion of diazotrophs scaled positively with community phosphorus use efficiency (Table 2). In particular, the proportion of *Nodularia* had a strong positive relationship with RUE_P_ (slope = 0.87; *P* = 7.4 × 10^−10^, *n* = 2279). The effect of *Aphanizomenon* was weaker, but still significant (slope = 0.24; *P* = 0.006, *n* = 2279). Unexpectedly, the relationship was also positive with nitrogen use, albeit not statistically significant (except for *Nodularia*, which was marginally significant; slope = 0.36; *P* = 0.01, *n* = 2162; Table 2).

**Table 2:** The linear relationship between resource use efficiency (RUE_P_ and RUE_N_) and diazotrophic proportion

	Total diazotrophs	*Nodularia*	*Aphanizomenon*
RUE_P_; *n* = 2279	0.31 ± 0.08; *P* = 10^−5^	0.87 ± 0.13; *P* = 10^−10^	0.24 ± 0.09; *P* = 0.006
RUE_N_; *n* = 2162	0.15 ± 0.08; *P* = 0.053	0.36 ± 0.14; *P* = 0.01	0.10 ± 0.09; *P* = 0.2

The parameter estimates are slopes of the linear regression components of the GAM models (±SE). The GAM model also accounted for the spatial dependence of RUE through a two‐dimensional smooth term of latitude and longitude. RUE was rescaled to standard scores to facilitate comparison between regression slopes.

As RUE was normalized to zero mean and unit standard deviation, we could directly compare the difference between the slopes. The slopes of RUE_P_ against *Nodularia* and *Aphanizomenon* were significantly different (*P* = 1.7 × 10^−6^), but only marginally different against RUE_N_ (*P* = 0.048). Further, the slopes of RUE_P_ and RUE_N_ against *Nodularia* were significantly different (*P* = 0.0067), but not against *Aphanizomenon* (*P* = 0.2).

We did not relate RUE with diazotroph biomass. A proxy of algal biomass (Chl *a*) is in the numerator of RUE formula, and the result would be trivial, overriding the niche separation effect between *Nodularia* and *Aphanizomenon*.

### Diazotrophs explain a small fraction of ambient phytoplankton community variation

**Figure 2:**
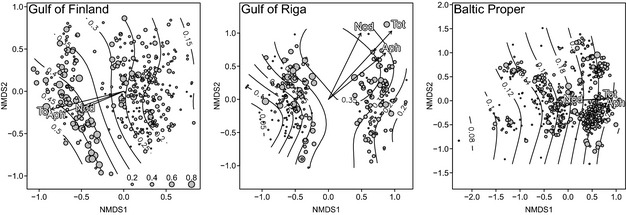
NMDS ordination plots of the non‐diazotrophic phytoplankton community (1994–2004) from the three main sub‐basins of the Baltic Sea. The symbol size reflects the proportion of total diazotrophs in the sample. The iso‐lines show the 2D response surface of the total diazotroph proportion. The arrows, superimposed on the ordination, show the direction and strength of diazotroph proportion (equivalent to the length of the arrow, numerical values and significance levels in Table 3).

Community analyses done with diazotroph biomass proportion and log‐transformed biomass agreed remarkably well, and only the proportion results are presented below. Unconstrained community ordination resulted in a structured distribution pattern of high bloom intensity samples on the ordination plane (Fig. 2). The gradient of diazotrophic biomass proportion on the ordination plane was weak (*R* ^2^ = 0.06–0.17), but statistically significant (Table 3). The correlation between *Aphanizomenon* and community ordination was consistently higher (*R* ^2^ = 0.05–0.13) compared to *Nodularia* (*R* ^2^ = 0.01–0.05).

**Table 3:** The strength of associations between diazotroph proportion and the variation of the ambient phytoplankton community in the main Baltic Sea sub‐basins

Sub‐basin	Analysis	Total diazotrophs	*Nodularia*	*Aphanizomenon*
GOR *n* = 259	aNMDS	0.08 (*P* < 0.001)	0.05 (*P* = 0.002)	0.05 (*P* = 0.002)
	bCCA	0.01 (*P* = 0.005)	0.01 (*P* = 0.005)	0.01 (*P* = 0.005)
	cADONIS	0.03 (*P* < 0.001)	0.01 (*P* < 0.001)	0.03 (*P* < 0.001)
BP *n* = 493	aNMDS	0.06 (*P* < 0.001)	0.07 (*P* = 0.01)	0.07 (*P* < 0.001)
	bCCA	0.005 (*P* = 0.005)	0.003 (*P* = 0.07)	0.004 (*P* = 0.005)
	cADONIS	0.01 (*P* < 0.001)	0.005 (*P* = 0.003)	0.02 (*P* < 0.001)
GOF *n* = 344	aNMDS	0.17 (*P* < 0001)	0.05 (*P* < 0.001)	0.13 (*P* < 0.001)
	bCCA	0.008 (*P* = 0.005)	0.007 (*P* = 0.005)	0.006 (*P* = 0.005)
	cADONIS	0.02 (*P* < 0.001)	0.007 (*P* = 0.003)	0.02 (*P* < 0.001)

GOR, Gulf of Riga; BP, Baltic Proper; GOF, Gulf of Finland; n, number of samples in each analysis.

Only samples from 1994 to 2004 are used. All significance levels (in parenthesis) were assessed by randomizing diazotroph proportions between the samples (999 simulations).

a Squared correlation coefficient (*R* ^2^) between NMDS ordination and the diazotroph proportion as an external variable.

b Constrained inertia in CCA ordination, that is the part of community variation, which can be explained by the diazotroph proportion.

c Community variation due to diazotrophs partitioned with permutational multivariate analysis of variance.

Constrained community ordination revealed that the diazotroph proportion explained a small fraction (< 1%) of the variation in community composition (Table 3). Although lower than the environmental fitting of diazotroph proportion on unconstrained ordination, the permutation test revealed that the constraints were statistically significant (*Nodularia* in the Baltic Proper being marginally significant).

Partial constrained ordination, where the effect of background environmental variables (irradiance, wind, year, day of the year and distance from shore) were conditioned out, changed the constrained variation only marginally, by a factor of 0.96–1.05 (data not shown). We therefore concluded that the detected community variation, ascribed to the proportion of diazotrophs, was indeed not due to the background environmental variables.

Permutational multivariate analysis of variance was in line with the ordination results and revealed that the proportion of diazotrophs did not explain more than a few percentage of the community variation (Table 3).

### Local similarity analysis reveals high proportion of positive and negative associations

**Figure 3:**
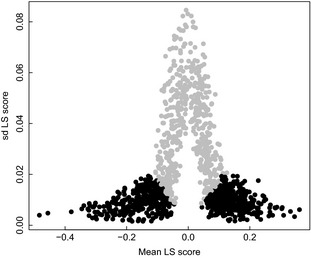
Standard deviations of the LS scores, as a function of the mean LS score. Results with total diazotrophs, *Nodularia spumigena* and *Aphanizomenon* sp., and all sub‐basins are pooled. Black symbols denote mean LS scores where all 1000 randomizations resulted in either negative or positive values, and are considered statistically significant in Table 4. Grey symbols denote LS scores with mixed signs. Note the high variability as the mean LS score approaches zero, designating species with neutral association with diazotrophs.

For LSA, we considered taxa, which were present in at least 10 samples per basin, resulting in community matrices with 161, 85 and 87 taxa for the Baltic Proper, Gulf of Finland and Gulf of Riga, respectively (Table 4). The average LS scores (mean of 1000 randomizations) ranged from −0.48 to 0.36. The standard deviation of the LS scores increased rapidly as the mean approached zero (Fig. 3), indicating that species with low mean scores had no apparent association with diazotrophs. We considered the mean LS score to be significantly different from zero when all the 1000 randomized estimates resulted in either positive or negative scores (Fig. 3). By using this conservative criterion, the proportion of significant associations ranged from 0.4 to 0.8 within the sub‐basins and diazotroph groups (Table 4).

**Table 4:** Significant associations and positive associations with diazotroph proportion and biomass (separated with ‘/’), as detected by local similarity analysis (LSA)

	Significant associations		Positive associations		
	Count^b^	Proportion^c^	Count^d^	Proportion^e^	*P*‐value^f^
GOF *n* a = 85					
Total diazotrophs	58/59	0.68/0.60	19/42	0.33/0.71	0.01/0.002
*Aphanizomenon*	56/57	0.66/0.58	19/40	0.34/0.70	0.02/0.004
*Nodularia*	36/46	0.41/0.46	15/25	0.43/0.54	0.5/0.66
GOR *n* a = 87					
Total diazotrophs	61/68	0.70/0.72	37/46	0.61/0.68	0.1/0.005
*Aphanizomenon*	67/68	0.77/0.72	42/46	0.63/0.68	0.05/0.005
*Nodularia*	53/61	0.61/0.64	33/40	0.62/0.66	0.1/0.02
BP *n* a = 161					
Total diazotrophs	88/103	0.55/0.6	57/90	0.65/0.87	< 0.01/< 0.001
*Aphanizomenon*	98/108	0.61/0.62	55/91	0.56/0.84	0.3/< 0.001
*Nodularia*	96/111	0.69/0.64	68/86	0.71/0.77	< 0.01/< 0.001

Sub‐basin codes as in Table 3.

a Total number of species analysed in each sub‐basin.

b The number of significant associations. Associations were conservatively considered significant when all 1000 re‐analysis with randomized sample order resulted in either positive or negative LS scores.

c The proportion significant associations.

d The number of positive associations.

e The proportion of positive associations out of significant associations.

f *P*‐value of the 1‐sample proportion test on equality of positive and negative associations.

To analyse whether the significant species‐specific associations with diazotrophs were predominantly negative or positive, we used 1‐sample proportions test with continuity correction. We found that the proportion of positive associations with diazotroph proportion was significantly higher than 0.5 in the Gulf of Riga and the Baltic Proper, and the proportion of negative associations was higher in the Gulf of Finland (Table 4). The statistical significance of these departures from equal proportionality was variable and without a clear pattern. In contrast, the proportion of positive associations with diazotroph biomass was always higher than 0.5. Except for *Nodularia* in the Gulf of Finland, the departures were statistically significant (Table 4).

**Figure 4:**
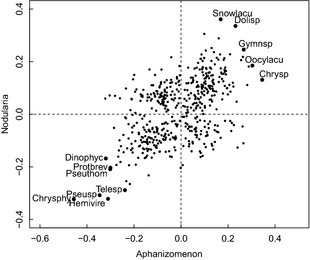
Relationship between the LS scores of *Aphanizomenon* sp. and *Nodularia spumigena*. The data points represent species. Species with strongest positive or negative co‐occurrence patterns (large symbols, labelled) are on the upper‐right and lower‐left parts of the data cloud. Chrysp – *Chrysochromulina* sp; Pseuthom – *Pseudopedinella thomsenii*; Dinophyc – Dinophyceae; Gymnsp – *Gymnodinium* sp; Dolisp – *Dolichospermum* sp; Protbrev – *Protoperidinium brevipes*; Pseusp – *Pseudopedinella* sp; Snowlacu – *Snowella lacustris*; Telesp – *Teleaulax* sp; Chrysphy – *Chrysophyceae*; Hemivire – *Hemiselmis virescens*; Oocylacu – *Oocystis lacustris*.

The overall positive correlation between the LS scores of *Aphanizomenon* and *Nodularia* (Pearson correlation coefficient 0.5; *P* < 10^−16^, *n* = 488; Fig. 4) indicates that species that had a positive or negative co‐occurrence pattern with one of the diazotroph species tended to have a similar pattern with the other cyanobacterium.

### Phylogenetic and trait‐based patterns were present in species associations with diazotroph biomass, but not with proportion

Overall, the species associations with diazotroph proportion were not significantly different from random expectation within the functional trait space or phylogenetic hierarchy, with the exception of functional trait convergence in the Gulf of Riga with species negatively associated with *Nodularia* (Table 5). In contrast, species associations with diazotrophic biomass were clearly non‐random in the Baltic Proper, and less so in the Gulf of Finland (Table 5). We did a one‐way analysis of variance to test whether there are differences in the association with diazotrophs between algal classes. The anova model indicated significant differences [*F*(8,134) = 4.53, *P* = 6 × 10^−5^] in the Baltic Proper, suggesting on average stronger positive associations within green algae, cryptophytes and other cyanobacteria, and negative associations with diatoms. This was also reflected in non‐random distribution of functional traits (Table 5). In particular, traits, which are phylogenetically conserved, like accessory pigment composition and silica requirement, were non‐randomly distributed among species with positive or negative association pattern with diazotroph biomass.

**Table 5:** Functional and phylogenetic similarity of taxa revealing positive (SES < 0) or negative (SES > 0) association with diazotroph cyanobacterial proportion and biomass (separated with ‘/’)

	Phylogenetic distance		Functional distance	
	SES	*P*	SES	*P*
GOF *n* a = 85				
Total	−0.3/0.9	0.4/0.16	0.80/−2.1	0.2/0.02
*Aphanizomenon*	1/1.4	0.2/0.08	−0.76/−2.1	0.2/0.02
*Nodularia*	0.2/1.1	0.4/0.14	−1.4/−1.1	0.09/0.14
GOR *n* a = 87				
Total	−0.5/0.56	0.3/0.3	1.2/−0.25	0.1/0.4
*Aphanizomenon*	0.3/0.49	0.4/0.3	0.14/−0.14	0.4/0.5
*Nodularia*	−1.7/−0.2	0.04/0.4	2.5/1	0.003/0.16
BP *n* a = 160				
Total	−0.3/4.3	0.4/0	−0.07/2.4	0.5/0.008
*Aphanizomenon*	1.7/4.3	0.04/0	1.3/2.85	0.1/0.002
*Nodularia*	−0.2/2.8	0.4/0.002	1.3/1.57	0.1/0.056

Sub‐basin codes as in Table 3.

a Total number of species analyzed in each sub‐basin. The statistical significance of the phylogenetic or functional effect size (expressed as *P*‐value) indicates the proportion of times when the observed MPD was lower (SES < 0) or higher (SES > 0) than the randomized MPD (9999 simulations).

## Discussion

The effects of diazotrophic cyanobacteria on natural phytoplankton communities are expected to be manifold, ranging from positive (new nitrogen supply to the planktonic ecosystem) to negative (toxic or other direct allelopathic effects, competitive advantage due to N‐fixation capacity). We therefore analysed the effects both from the resource use perspective and through direct associations with co‐occurring phytoplankton taxa.

### RUE patterns relate to niche differentiation in P acquisition

Diazotroph proportion was positively related to the community phosphorus use efficiency (RUE_P_). Contrary to our expectation, there was no concomitant negative association with the nitrogen use efficiency (RUE_N_). Diazotrophs are postulated not to be N‐limited, and when in abundance, may shift the otherwise N‐limited plankton community to transient P limitation. Indeed, in the Baltic Sea, seston C:N ratios in the filamentous cyanobacteria size fraction have suggested N sufficiency during the late summer cyanobacterial bloom season, whereas the C:P ratios indicated P limitation (Degerholm *et al*. 2008). Once dissolved inorganic P pools and internal cellular storages are exhausted, cyanobacteria compete with other phytoplankton for P (Vahtera *et al*. 2010). P competition can further be aggravated by the release of the newly fixed N, relaxing N limitation at both, cellular and community levels (Nausch, Nausch & Wasmund 2004; Vahtera, Laamanen & Rintala 2007). As phosphorus becomes progressively scarce, increasing P use efficiency along with the build‐up of a cyanobacterial bloom is a due expectation and was indeed substantiated with field data. We concomitantly expected a decrease in RUE_N_, which could reflect the relaxation of N limitation by the input of new nitrogen into the system. This expectation was not, however, confirmed with evidence. On the contrary, also RUE_N_ showed a positive relationship with diazotrophs, albeit not always statistically significant.

Clearly, there are also other factors that affect community RUE. Firstly, even during extensive cyanobacterial bloom events, the non‐diazotrophic phytoplankton community can dominate both biomass and primary productivity and remain N‐limited despite new nitrogen flux via diazotrophs. Although a large fraction of the newly fixed N_2_ is exudated in a bioavailable from on a daily basis, the measured total rates of N fixation can contribute only a modest fraction (< 20%) to the amount of N needed for the community primary production during bloom events (Ohlendieck *et al*. 2007). Therefore, potential relaxation of community N limitation by N fixation may remain hardly noticeable.

Second, in the Baltic Sea, there is a high background of refractory dissolved organic nitrogen (Asmala *et al*. 2013), which also is accounted for in the calculation of RUE_N_. When new bio‐available nitrogen enters the system, for example through exudation of fixed nitrogen, the proportion of the refractory pool diminishes, leading to an overall increase in N use efficiency, and effective decoupling between RUE_N_ and N limitation. Therefore, cyanobacterial nitrogen fixation can induce multiple mechanisms on nitrogen use efficiency, which partly compensate each other. The empirical net effect can therefore be no change or even increase of RUE_N_, as was found in this study.

*Nodularia* had a significantly stronger scaling with RUE_P_, which is in line with its experimentally observed use of dissolved organic P and higher phosphate affinity compared to *Aphanizomenon* (Larsson *et al*. 2001; Vahtera, Laamanen & Rintala 2007), and reflects a niche differentiation in phosphorus acquisition between the two species. Increasing *Nodularia* proportions could thus drive the community towards more severe P limitation, and correspondingly to higher P use efficiency compared to *Aphanizomenon*‐dominated communities. The growth of *Aphanizomenon* depends firstly on external phosphate supply (Vahtera, Laamanen & Rintala 2007), which is ample early during the season, after occasional upwelling pulses in frontal regions (Kononen *et al*. 1996), as well as in deeper layers close to the nutricline. Accordingly, field studies have indicated vertically separated distribution of the two species, with *Aphanizomenon* having a deeper biomass maximum than *Nodularia* (Hajdu, Höglander & Larsson 2007).

This vertical segregation, caused by a combination of buoyancy regulation with gas vacuoles and physical mixing of the water column, is supported by the lower temperature and light optima of *Aphanizomenon*, compared to *Nodularia* (Lehtimäki *et al*. 1997). When phosphate supply is plentiful, *Aphanizomenon* accumulates intracellular P reserves, which are estimated to be sufficient for up to a ten‐fold increase of cellular biomass (Larsson *et al*. 2001; Kangro *et al*. 2007). Unlike *Aphanizomenon*,* Nodularia* seems to lack the capacity to grow extensively on internal P reserves and must therefore continuously obtain P from external sources (Larsson *et al*. 2001).

As the blooms develop during the regenerated production period with mostly non‐detectable concentrations of inorganic P, these sources must originate from the ambient microbial food web, mainly from bacterial decomposition in the favourable microenvironment of *Nodularia* aggregations (Tamminen 1989). These two dominant diazotrophic species can thus be considered as a storage specialist and an affinity specialist, respectively, which are essential determinants of their successful bloom‐forming strategies and niche partitioning, despite their apparently similar functional roles in overall aquatic biogeochemical cycles.

### Blooms have no consistent or predominantly negative effect on ambient phytoplankton community

Diazotrophic cyanobacteria had a low, but clearly detectable, covariation with the non‐diazotrophic component of the phytoplankton community (Table 3). A small, but statistically significant effect was indicated by all the analysis methods: fitting the diazotroph proportion on unconstrained ordination space, ordination constrained by the diazotroph proportion and permutational multivariate analysis of variance. On a species level, over half of the ambient non‐diazotrophic phytoplankton species revealed statistically significant associations with diazotrophs, either positive or negative. These associations had no consistent trait‐based or phylogenetic pattern, except in the Baltic Proper, when species associations with diazotroph biomass were assessed.

In contrast to the common perception and a majority of literature reports (see an overview by Karjalainen *et al*. 2007), we cannot therefore confirm that the effect of diazotrophs on other phytoplankton species was predominantly negative. Overall, in the Baltic Proper and the Gulf of Riga, there were significantly more species with a positive association than with negative association with increasing diazotrophic proportion. The positive association pattern was predominant and consistent over all basins when diazotroph biomass was considered (Table 4). LSA is a correlative analysis and makes no assumption on the causality of the associations, neither does it point to any particulate mechanism, direct or indirect. In particular, species associations with high diazotroph biomass are more difficult to interpret. These may reflect species adaptations to environmental conditions pertinent to intense cyanobacterial blooms, such as high temperature and stratification, masking the direct effect of diazotrophic cyanobacteria. Allelopathic effects are probably important determinants of community composition (Suikkanen 2004; Engström‐Öst *et al*. 2011), but we believe our observed pattern of positive and negative associations may reflect transient segregation along nutrient supply ratio, in line with the resource competition theory (Tilman 1977). This is also supported by the positive correlation between species associated with *Aphanizomenon* and *Nodularia* proportions (Fig. 4).

Negative impact of diazotrophic cyanobacterial blooms on selected zoo‐ or phytoplankton groups has been shown earlier in numerous experimental studies (Koski, Engström & Viitasalo 1999; Suikkanen 2004; Engström‐Öst *et al*. 2011), while positive or neutral effects easily circumvent reporting (but see Hogfors *et al*. 2014). Here, we have taken a more holistic approach, to consider the consequences on ambient non‐diazotrophic phytoplankton community as a whole in the fully natural environment, counteracting the possible publication bias of reporting predominantly negative effects in experimental studies.

The 7000‐year history of cyanobacterial accumulations in the Baltic Sea has selected for strains, which are ecologically highly efficient in the current geological stage of the Baltic Sea. The dominant cyanobacterial bloom species share the diazotrophic N acquisition strategy, but differ fundamentally in P acquisition as well as other autecological traits (Larsson *et al*. 2001; Vahtera, Laamanen & Rintala 2007). The relatively slow growth rates (Lehtimäki *et al*. 1997; Vahtera, Laamanen & Rintala 2007) are effectively compensated by lack of N limitation and resistance to herbivory (Sellner, Olson & Olli 1996), resulting in high ecological fitness and biomass build‐up, but at the same time a dead end in the grazing food chain. Their population dynamics thus seem to follow own trajectories, essentially uncoupled from the ambient non‐diazotrophic plankton community. As a result, the covariation between the ambient phytoplankton community and the diazotroph assemblage is low.

### N fixers in the planktonic community

Cyanobacterial N_2_ fixation is an important part of the marine nitrogen cycle (Canfield, Glazer & Falkowski 2010). It provides a source of new nitrogen (as opposed to N that is regenerated from existing organic material) in surface waters that can support biological carbon export and sequestration (Deutsch *et al*. 2007). However, many questions remain about marine N_2_ fixation, particularly, the role and proportion of different functional types of diazotrophs in the total N_2_ fixation, as well as the potential for heterotrophic N_2_ fixation. The role of diazotrophs in the assembly of the rest of the phytoplankton community, and the community resource use efficiency, has not been addressed before.

Here, we have focused on heterocystous filamentous cyanobacteria, the only type of diazotrophs, which is easily recognizable by morphology. In many ways, *Nodularia* in the Baltic Sea can be seen as a surrogate of *Trichodesmium* in the tropical oceans. Both benefit from elevated temperature, light and stable water column, are affinity specialists with respect to phosphate and can utilize dissolved organic P by expressing alkaline phosphatases and release substantial fixed N to the environment (Lehtimäki *et al*. 1997; Wannicke, Koch & Voss 2009). Their filamentous aggregates are easily observed and collected and are the reason why other groups of cyanobacteria did not previously receive much attention.

Nitrogen fixation by small unicellular cyanobacteria has been known for over a decade (Zehr *et al*. 2001; Moisander *et al*. 2010), and when at high density, they could add as much new nitrogen as *Trichodesmium* (Sohm, Webb & Capone 2011). The oceanic unicellular diazotrophs have a wide temperature range (Needoba, Foster & Sakamoto 2007), which further raises the question whether this type of diazotrophs can be of importance during the cooler season in the Baltic Sea. Community DNA screening has not revealed the presence of small‐sized diazotrophic cyanobacteria in the Baltic Sea (Farnelid, Öberg & Riemann 2009). However, high rates of nitrogen fixation by heterotrophic and photoheterotrophic bacteria have been recently measured in the Baltic Sea (Bentzon‐Tilia, Traving & Mantikci 2014), which could potentially cause additional variation in the community response to heterocystous cyanobacteria in our analysis. The role of heterotrophic N_2_ fixation remains speculative until the distribution and identity of these organisms is better known. Previous size‐fractionated studies have shown that organisms < 5 μm contribute very moderately to overall N_2_ fixation in the Baltic Sea (Ohlendieck *et al*. 2007). We therefore believe that our results represent a fair approximation of the effect of pelagic nitrogen fixers on the community assembly and resource use efficiency.

Our results are in line with previous studies showing that high rates of N_2_ fixation drive phytoplankton community to severe P limitation (Wu *et al*. 2000). We further discriminated between the two dominant diazotrophs, showing that *Nodularia* dominance leads to higher community P use efficiency compared to *Aphanizomenon*, which is in line with the niche differentiation in P acquisition – the former being uptake specialist and the latter storage specialist. However, given the high rates of N_2_ fixation and cellular leakage of fixed N to the environment (Larsson *et al*. 2001; Mulholland 2007; Ohlendieck *et al*. 2007), it was unexpected to find only a modest effect of the nitrogen enrichment on the ambient phytoplankton community variability.

## Data accessibility

Community matrix, sample table, phytoplankton functional traits and phylogeny data: Dryad Digital Repository http://dx.doi:10.5061/dryad.d0826.
